# Sleep health in black families: bedtime routines and sleep patterns among young children

**DOI:** 10.3389/frsle.2026.1876293

**Published:** 2026-07-24

**Authors:** Alicia Chung, Monica Stanton-Koko, Willa Johnson, Azizi Seixas, Debbie Chung, Iheoma U. Iruka, Laurie Brotman, Keng-Yen Huang, Girardin Jean-Louis

**Affiliations:** 1 Center for Early Childhood Health and Development, Department of Population Health, NYU Grossman School of Medicine, New York, NY, United States; 2 Health Studies Department, Borough of Manhattan Community College, City University of New York, New York, NY, United States; 3 John Jay College, City University of New York, New York, NY, United States; 4 Center for Translational Sleep and Circadian Sciences, Department of Psychiatry and Behavioral Sciences, University of Miami Miller School of Medicine, Miami, FL, United States; 5 Department of Informatics and Health Data Science, Medicine, Miami, FL, United States; 6 Department of Psychiatry and Behavioral Sciences, University of Miami Miller School of Medicine, Miami, FL, United States; 7 Department of Maternal Child Health, University of North Carolina Gillings School of Global Public Health, Chapel Hill, NC, United States; 8 Department of Psychiatry, NYU Langone Health, New York, NY, United States

**Keywords:** bedtime routines, mixed-methods, pediatric sleep, qualitative and quantitative study, sleep health disparities

## Abstract

**Introduction:**

Young Black children experience poorer sleep health than children from other racial/ethnic groups. Tailored family-centered interventions are needed to improve sleep health among young Black children. Tailoring requires information about parent perceptions, practices and context. The purpose of this study was to (1) understand the contextual definition of sleep health for Black families; (2) bedtime routines and sleep practices among Black families; and (3) parent preferences that may inform intervention design.

**Methods:**

We engaged 30 Black parents of 3–8-year-old children with mild sleep problems (e.g., child takes >30 min to fall asleep at bedtime) in this study. Parents completed three ratings scales about sleep health practices and child bedtime behavior and a semi-structured interview. Statistical analysis included descriptive statistics and correlations. We used an implementation science rapid qualitative analysis approach to analyze qualitative data from the interviews.

**Results:**

The final sample identified as 52% African-American, 14% Jamaican, 10% Haitian, and 26% mixed multi-ethnic groups. Parent mean age was 41 years and child mean age were 5 years old. About 80% of responders were women. About one-third of the sample held a doctoral degree, and half the sample had a bachelor's or Master's degree. Parents answered 56% of sleep knowledge questions accurately (ranging from 32 to 100%) and reported an average of two sleep problems. Parents described healthy sleep as involving flexibility in sleep timing and bedtime routines. More than half of parents reported co-sleeping practices, with reasons ranging from a strategy to address night wakings, to a way to preserve bonding. Later child bedtime (after 9 pm) was associated with bedtime resistance, permissive parenting, and parent stress. Many parents reported that they had poor sleep quality and duration.

**Discussion:**

Findings elucidate a range of factors to consider in tailoring sleep health interventions for Black families of young children. These include the common practice of co-sleeping, parents' value for flexibility related to bedtime and bedtime routines, and parents' own challenges with getting enough and good quality sleep. Tailored family-centered interventions will benefit from considering these factors.

## 1 Introduction

Sleep is critical for healthy growth and development (Matricciani et al., 2019; Zhou et al., 2015). Adequate sleep during early childhood promotes healthy weight status, academic achievement, and mental wellbeing (Carter et al., 2014; Felso et al., 2017; Gregory and Sadeh, 2012; Williamson et al., 2020). The American Academy of Sleep Medicine recommends that children 3–5-year-olds obtain 10–13 h of sleep in a 24-h period, including naps (Paruthi et al., 2016). More than one-third of young children do not get sufficient sleep (The White House, 2025). Poor sleep health places children at risk for a range of metabolic conditions, including obesity and diabetes (Centers for Disease Control Prevention, 2024). Establishing a regular bedtime routine and consistent sleep timing have been identified as two key practices associated with early childhood sleep health (Matricciani et al., 2019; Miller et al., 2014; Mindell and Williamson, 2018; van Schalkwijk et al., 2015).

Healthy sleep is defined by duration, quality, efficiency, regularity, and circadian alignment (DelRosso, 2025). In children, healthy sleep is marked by capacity for independent sleep, absent of night wakings that require parental assistance or children cannot fall back asleep, obtaining adequate sleep duration (recommended by age group), and supported by a consistent bedtime routine (Bacaro et al., 2019; Suni and Vyas, 2025). Communal culture among Black and other non-white populations may shape what healthy sleep looks like (Schwartz et al., 2010). Sleep health in early childhood is influenced by the lived experiences and values of parents and other caregivers (e.g., grandparents). This may lead to the inclusion of multiple family members (e.g., mother, father, grandmother) being a part of evening routines or co-sleeping (e.g., room or bed sharing). Household structure (e.g., multi-generation housing) and context (e.g., resources) may also contribute to co-sleeping practices. Differences in sleep patterns and sleep problems are well documented in Black children (Williamson et al., 2017). Our study aims to begin to provide a picture of what sleep health looks like for Black families, starting with a sample of parents interviewed mostly from the New York tri-state area.

Short sleep duration among Black children is 53% compared to 28% of white children (Centers for Disease Control Prevention, 2024). Although there are some studies that provide information on sleep disparities prevalence rates and sleep patterns (Guglielmo et al., 2018; Smith et al., 2019), the authors call for an understanding of the underlying causes of sleep disparities, including parent beliefs about sleep, typical sleep practices, and an examination of the factors that contribute to these disparities (Guglielmo et al., 2018). In response to this call to action, qualitative data is needed to listen and learn from Black parents of young children. Our qualitative and quantitative study aimed to understand the contextual factors that affect sleep, parent beliefs about their child's sleep, typical bedtime routines and sleep practices through a qualitative and quantitative approach. Our qualitative and quantitative approach will fill this gap in the literature, and fulfill the goal of providing a contextual definition of sleep health among Black families, to secondarily inform intervention design. To this end, primary data collection began with understanding the lived experience of Black mothers during their child's evening bedtime routine.

Research shows that Black women, who are often the primary parent for young children, may face an undue amount of stress (Erving et al., 2024). The added burden of taking on multiple caregiving responsibilities, referred to as the Superwoman schema, is associated with cardiovascular health problems among Black women (Erving et al., 2024). Stress may affect a mother's capacity to support bedtime routines, set consistent bedtimes and engage in evidence-based strategies shown to support child sleep health (McQuillan et al., 2019). Black families across the economic strata are more likely to face discrimination and other stressors, that contribute to sleep problems (Jackson et al., 2013). Black families with low incomes may experience additional stressors associated with limited resources and other contextual factors that affect child sleep (Covington et al., 2019). The specific ways that parent stress influences bedtime routines and child sleep health across a range of life circumstances remain unclear (Bacaro et al., 2019; McQuillan et al., 2019; Teti et al., 2010). Our study employed a human-centered design approach, including semi-structured empathy interviews, to understand parent emotional affect as they go through each step of their child's bedtime routine.

This study aimed to address knowledge gaps through a qualitative and quantitative study. The objectives of this study were to explore (1) understand the contextual definition of sleep health for Black families; (2) bedtime routines and sleep practices among Black families; and (3) parent preferences that may inform intervention design.

## 2 Methods

Data collection for this study was conducted from February to September 2024.

### 2.1 Participants

Eligibility criteria included being a self-reported Black parent aged 18 or older, who is English-speaking, and has a young child (3–8 years old) experiencing sleep problems (as reported by the parent). Interested parents completed a set of screening questions in RedCap from a QR code link on a study flier. After meeting this initial eligibility, a study team member reviewed consent with potential participants and determined final eligibility. The study included a total of 30 participants. Participants were compensated $50 for completing both the semi-structured interview and survey.

### 2.2 Procedures

We recruited participants in person at local community-based organizations, such as libraries, child-care centers and faith-based organizations, in New York City, Mount Vernon and Yonkers neighborhoods with high concentrations of Black families. We also recruited families online by posting an IRB-approved recruitment flier on social media platforms (e.g., Facebook, Instagram, LinkedIn, and WhatsApp) on pages and groups specially geared toward Black parents across the United States. Additionally, we used a snowball recruitment procedure where existing community partners and networks shared the flier with potentially eligible families.

Participants completed a 60-min semi-structured interview and a survey. Interviews were conducted in person or virtually via WebEx/Zoom and were audio-recorded. The recordings were stored securely and transcribed. All interviews were recorded with the interviewees' consent and transcribed afterwards. All procedures were approved by the university IRB (i23-00532).

Qualitative data from parents provided illustrative insights that enhanced what we know about the contextual factors in Black homes during bedtime routine hours, using Microsoft Excel software to identify codes and conduct an inductive thematic analysis to collate emerging themes. Quantitative data include parent self-report items from three validated measures on child routines and parenting behaviors related to child sleep.

### 2.3 Semi-structured interviews

We conducted interviews in person or via zoom to best accommodate the parents' comfort level or geography. Semi-structured interview questions were guided by the socio-ecological model of sleep, and included questions on household context, environment, and family structure, as well as beliefs about child sleep health and current sleep practices (Grandner, 2019). We presented a range of evidence-based strategies from several programs, including the Sleep Train program (McQuillan et al., 2023, 2017), the ParentCorps routines chart (Dawson-McClure et al., 2022; ParentCorps News [Online], n.d.), and a selection from the audio-based sleep-aide, Moshi (Chung et al., 2022), to obtain parents' feedback about existing evidence-based strategies.

Semi-structured interviews focused on understanding the three study objectives listed in Table 1. For instance, to understand Objective 1-healthy sleep definition for Black families, we asked “What does an ideal night sleep look like for you?” “It's around 6 pm, what does that typically look like for you and your family?” (listening for signs of parent stress, understand sleep context/evening routine before bedtime)? “Describe what the bedtime routine looks like for you and your child.” “Are other parents/caregivers involved in the bedtime routine (listening for bedtime resistance, routine practice, co-sleeping)?” “Describe any sleep challenges (listening for sleep quality, sleep timing, parent sleep problems, night waking and morning wake time challenges).” After each response, we asked interviewees to pause and reflect on how they were feeling. To understand Objective 2-Bedtime routines and sleep practices among Black families, quantitative measures completed by parents. And Lastly, to understand Objective 3, parent preferences that may inform intervention design, parents provided feedback about existing evidence-based sleep strategies, as well as completed a quantitative survey.

**Table 1:** Study objectives with measure and sample questions.

Objective	Quantitative measure (s)	Qualitative question examples
1. Understand the contextual definition of sleep health for Black families		Sample question: 1. Can you walk me through your day from start to finish? What are you feeling?
2. Bedtime routines and sleep practices among Black families.	Parent-child sleep interaction and bedtime routine questionnaires	
Sample questions:	1. My child sleeps in my room all night. 2. My child comes to my room at bedtime. 3. During weeknights for the past month, how often did your child perform the same activities in the hour before going to bed (for example, bathe, brush teeth, read/listen to story)?	1. What does getting your child to sleep look like in your home? It's around 6pm..what does that look like for you? How do you feel after that? 2. What do some of the more challenging nights look like when getting your child to bed? Can you describe it to me please?
3. Parent preferences that may inform intervention design	Child routines inventory	
Sample questions:	1. My child takes turns with family members talking about their day. 2. My child has a set routine for getting ready in the morning (e.g., brushing teeth, washing face, doing hair and dressing). 3. My child hugs/kisses family member before bed. 4. My child wakes up at about the same time on weekdays. 5. My child has a set routine for getting ready in the morning (e.g., brushing teeth, washing face, getting dressed).	1. How might we create the optimal environment for your child to sleep? 2. What does your child's sleep environment look like? Is it the same or different each night? 3. Are there other adults in the home during bedtime hours? If so, what role do they play in the process of getting your child to bed? 4. Do you have any stress that affect you when you get home? Do these stressors affect you during your child's bedtime?

### 2.4 Sleep practice and context quantitative survey measures

Parents completed three surveys, Parent-Child Sleep Interaction Scale (PSIS) (Alfano et al., 2013); Bedtime Routines Questionnaire (BRQ) (Henderson and Jordan, 2010) and Child Routines Inventory Scale (CRI) (Sytsma et al., 2001). We also included 9 additional true/false questions on sleep health knowledge, benefits of healthy sleep, sleep timing, independent sleep, 2 multiple choice questions on family structure and 2 multiple-choice questions about sleep timing consistency. In total, parents responded to 75 items that captured 16 constructs (59 items from the 3 surveys).

We used three subscales from the *Parent-Child Sleep Interaction Scale* (Alfano et al., 2013). That capture sleep practices: *sleep Reinforcement, which assess praising and reassuring positive sleep behaviors (5 items*, α = *0.65 using this sample; “I praise my child for good sleep behaviors”; “I reassure my child about his/her ability to fall/stay asleep”); Sleep Conflict, which capture bedtime resistance* (4 items; α = 0.71 using this sample, “My child and I argue about bedtime/sleep schedules”; “At bedtime, I remind/tell my child several times to go to sleep”) and *Sleep Dependence, which capture co-sleeping/bedsharing …* (3 items; α = 0.75 using this sample; “My child comes to my room at bedtime”; “My child sleeps in my room all night”; and our added question “My child sleeps in my bed all night”). Parents report on a 5-point scale (0 = Never, 2 = Sometimes, 4 = Always/Almost Always), ranging from “Never” to “Always/Almost Always.” Higher scores reflect more problematic bedtime resistance, sleep dependence and sleep reinforcement interactions.

The *Bedtime Routines Questionnaire* (Henderson and Jordan, 2010), a 31-item parent-reported measure, including 8 subscales (consistency, including routine behaviors and environment, reactivity, and adaptive and maladaptive behaviors); alpha ranging from 0.69 to 0.86 with the study sample) and captures consistency of weekday and weekend bedtime routines and adherence to ideal bedtime practices. Parents respond to items on a 5-point scale, (1 = Almost Never; 3 = Half the time; 5 = Nearly Always), ranging from “Almost Never” to “Nearly Always.” Sample items include: “during weeknights, how often did your child perform the same activities in the hour before going to bed?”; adaptive behaviors (e.g., story, bath, tucked-in); and maladaptive behaviors (e.g., active play, tv before bedtime). We added an item to maladaptive behaviors asking about iPad use before bedtime.

The *Child Routines Inventory* (Sytsma et al., 2001), included 20 items (2 subscales: daily living routines and discipline routines, alpha range 0.74–0.78) parent rating scale that assesses children's daily living and discipline routines. Household responsibilities and homework routine subscales were omitted to reflect age-appropriate tasks for the target age group. Parent responses were on a 5-point scale, (0 = Almost Never; 2 = Somewhat; 4 = Nearly Always) ranging from “Almost Never” to “Nearly Always.” The option “Prefer not to respond” was also included. Sample items included, “My child takes turns with family members talking about their day;” “My child hugs/kisses family member before bed”; and “My child spends time talking with parent each day.” An example of a modified item for the CRI Scale included changing the following item from, “My child is disciplined for misbehavior (e.g., time out, loss of privilege or spanking),” to “My child is disciplined for misbehavior (e.g., loses playtime, something he/she enjoys is taken away.” All measures demonstrated good reliability, with correlation coefficients ranging from 0.69–0.86.

### 2.6 Statistical analysis

We analyzed quantitative data from questionnaires using descriptive statistics (frequencies and percentages for categorical variables and means and standard deviations for continuous outcomes). Descriptives and correlation testing was used to compare differences across groups by socio-demographics (e.g., education level, child sex). A composite variable of healthy sleep was generated based on parent responses to the child bedtime items (e.g., 9 pm or earlier bedtime, sleep onset < 15 min). IBM SPSS Statistics Version 28.0.1.1 (14) software was used for our quantitative analysis of descriptive statistics, mean scores and Person's r correlations. All 30 interviewees completed the survey component in full, except for one participant who did not complete the surveys, resulting in a sample size of 29/30 or 97% completion rate for the quantitative component.

### 2.7 Qualitative analysis

Phenomenology was applied to understand the lived experience of Black parents with young children and the concept of contextual factors that contribute to children's bedtime routines and bedtimes (Creswell, 1998). Given the use of phenomenology for our study, a study sample of 30 participants was more than sufficient. The study team (five members) convened bi-weekly to code and develop matrix summaries. A matrix, a streamlined format for displaying the summary points for the purpose of analysis, allowed for identification of similarities, differences and trends across groups (e.g., respondent x domain), was created in Microsoft Excel (Averill, 2002) We coded qualitative interviews and analyzed data using an implementation science rapid qualitative analysis approach (Hamilton et al., 2023). This qualitative analysis approach was applied to understand context and tailoring of implementation for sleep strategies. This approach included the development of transcript summaries of what interviewees said and put into sub-categories, that we later put into domain names based on group consensus and interpretation, domain conversion and analysis, group pattern recognition and matrix incorporation and concision among team members. Lead author, AC, conducted most of the interviews and co-author, WJ, led the matrix development.

We summarized interviews by converting content into key bullet point phrases under each domain in the matrix spreadsheet. We created transcript summaries and organized them by interview ID number, sleep strategy review, and household factors revolving around the child's sleep. We calculated the number of family bedtimes reported across all the interviews. We further refined the matrix summaries, consolidating key insights related to notable mentions and primary info regarding the sleep behavior, environment, struggles, and interventions reactions. We also discussed sleep strategy reactions, quantified and ranked them in terms of acceptance and use among Black families.

## 3 Results

Table 2 provides a description of the study sample characteristics. Among the 30 parents interviewed, 52% identified as African-American, 14% Jamaican, 10% Haitian, and the remainder West Indian, West African or mixed multi-ethnic groups. Parents were 41 years old on average and children were about 5 years old. About 80% of responders were women. Education level skewed high, with one third of the sample holding a doctoral degree, and half the sample holding a bachelor's or Master's degree.

**Table 2:** Descriptive demographics of the study sample.

Children (*n* = 29)	Mean (SD)/*n* (%)
Age	4.76 (1.65%)
Male	16 (55%)
Children with a sibling	19 (65.6%)
Parents (*n* = 29)	
Age	41 (4.39%)
Female	24 (82.8%)
Married	20 (69.0%)
Race/ethnicity	
White	1(3.3%)
Black	28 (96.7%)
Parent origin/ancestry	
African-American	15 (51.7%)
Jamaican	4 (13.8%)
Haitian	3 (10.3%)
Nigerian	1 (3.4%)
Barbadian	1 (3.4%)
West Indian	1 (3.4%)
Multiple ethnicities	2 (6.9%)
Not listed	1 (3.4%)
Education	
Some high school	1(3.4%)
Occupational/technical program	1 (3.4%)
Associate's degree	1 (3.4%)
Bachelor's degree	5 (17.2%)
Master's degree	12 (41.4%)
Doctoral degree	9 (31%)

### 3.1 Quantitative results

As shown in Table 3, more than half of the sample experienced more than one sleep problem (55%). All families reported having a bedtime routine. Most reported their child's bedtime at 9 pm or earlier (82%), less than 15 min for sleep onset (86%), and consistent wake times on 5 or more days per week (93%). Just over half the parents (56%) reported that they shared a bed or room with their child. Table 4 includes results of parent responses to sleep knowledge questions. Parents were accurate on 56% of the knowledge questions about child sleep. Parents gave incorrect responses to questions about the importance of bedtime consistency, benefits of healthy sleep, and recommended sleep duration for school-aged children (5–8 years old).

**Table 3:** Child and parent sleep patterns and sleep practices.

Child sleep pattern	*n*/(%)	
>1 sleep problem	16 (55%)	
Have a bedtime routine	29 100%	
Co-sleep with a parent (share a bed or room)	16 (56%)	
Child bedtime		
9 pm or earlier	24 (82%)	
After 9 pm	6 (18%)	
Sleep onset (< 15 min)	25 (86%)	
Consistent wake time (>5 nights)	27 (93%)	
Parent sleep pattern	No	Yes
Got enough sleep	76%	24%
Good sleep quality	66%	34%

**Table 4:** Parent sleep knowledge and belief questions.

Question	% correct	% incorrect
Children should have the same bedtime and waketime on weekdays and weekends.	55.2%	44.8%
Children only need a bedtime routine if they are having trouble falling asleep.	100%	0%
Well-rested children do not need an alarm clock to wake up in the morning.	46.4%	53.6%
Children who do not get enough sleep are more likely to be underweight than overweight.	44.8%	44.8%
Children who do not get enough sleep are more likely to have poor behavioral problems.	93.1%	6.9%
Children who do not get enough sleep are more likely to have poor school performance.	96%	3.4%
Snoring indicates that a child is sleep well.	100%	0%
The average preschooler (3–4 years old) needs about 10 h of sleep per 24 h.	86.2%	13.8%
The average school-aged child (5–8 years old) needs about 8 h of sleep per 24 h.	32.1%	67.9%

Parents also reported on their own sleep patterns. The majority (76%) reported that they do not get enough sleep, and 66% reported poor sleep quality (Table 3). Nearly all (90%) parents reported feeling exhausted or tired after putting their child to sleep, 17% felt overwhelmed, and 23% felt frustrated. Approximately 43% reported feeling work related stress and stress balancing work and parenting, while 17% reported experiencing financial stress. Other stressors included, routine getting thrown off, managing daily tasks/chores, child misbehavior at school and at home, not getting a break throughout the day, single parenting, caring for aging parents, child technology usage, divorce/separation, child medical issues, or a racist incident at school.

Results from the Bedtime Routine Questionnaire indicated that frequent bedtime routines were correlated with more independent sleep, less conflict at bedtime, and more daily routine. Bedtime routines were related to higher daytime alertness, less tantrums, better mood and sleep. Parents' use of positive reinforcement/praise of child was significantly correlated with both adaptive bedtime behaviors (e.g., bath, story) (*r* = 0.39, *p* = 0.04) and maladaptive behaviors (e.g., screen time before bed) (*r* = 0.39, *p* = 0.03).

Results from the Child Routine Inventory indicated that daily living routines (morning and evening) were a common practice in the home. The mean scores for daily living routines and discipline routines were 3.4 and 3.0 respectively. Bedtime resistance (*r* = 0.54, *p* = 0.003) and permissive/gentle parenting (*r* = −0.531, *p* = 0.004) were significantly correlated with later bedtimes. Daily living routines (e.g., morning and evening routines) were significantly correlated with bedtime routine adaptive behaviors (*r* = 0.434, *p* = 0.019); and independent sleep (e.g., child sleeping in their own bed. (*r* = −0.44, *p* = 0.017). Lastly, higher rates of bedtime routines were associated with greater independent sleep (*p* < 0.001).

Parent-Child Sleep Interaction Scale mean scores were 1.4 for sleep dependence, 1.7 for sleep conflict, and 2.1 for sleep reinforcement. Lower scores indicated lower frequency of occurrence (e.g., 5-point scale (0 = Never, 2 = Sometimes, 4 = Always/Almost Always). Co-sleeping was significantly correlated with bedtime resistance (*r* = 0.384, *p* = 0.04).

### 3.2 Qualitative results

#### 3.2.1 Contextual definition of sleep health: parent perceived ideal night sleep

When asked, “What would an ideal night routine look like for you?” Parents spoke of being able to get children to bed earlier, closer to 8 pm, with easier transition between tasks and settling in from after-school and work activities. Aspirational bedtime routine goals seemed to include a less stressful process that was more seamless and easily flowed right into the child sleeping, reduced bedtime resistance, and without the use of medication. A few select quotes from parents are as follows: “* I end up working longer than I'm supposed to and then dinner gets delayed and it's a whole thing. So, but* ***my ideal would be starting the*** ***routine earlier, more like eight, um then the normal*** *stuff that I just mentioned.”*

“*I don't know. Something to help kids naturally sleep. …****Not medicated to go to*** ***sleep*.**”

“*… When I say OK, quiet at that time and he runs in his bed and he goes to sleep*, ***he*** ***doesn't keep on like negotiating with me about time and giving him, giving him more time*** ***to play or to do something else****.”*

#### 3.2.2 Parent sleep practices-bedtime routines, sleep timing, co-sleeping

Parents shared typical evening routines commencing after school, care, or activity pick-up. These generally included homework, dinner, free time, a bath or shower, brushing teeth, storytime, prayer, and bedtime. During the interviews, about 30% indicated bedtime between 7 and 8 pm, 40% between 8 and 9 pm, and 30% between 9 and 10 pm. These responses to interviews suggest that more families have later bedtimes than reported on the surveys (18% reported bedtimes after 9 pm). Parents expressed overwhelming exhaustion after putting their child to sleep, a need for flexibility with bedtime routine start time and components of the routine (e.g., moving bathtime to morning instead of evening), and a desire for connection and quality time with their child.

Half of the sample reported co-sleeping behaviors. Parents described co-sleeping behaviors as being related to children seeking comfort from parents in bed, getting into their child's bed to help them to fall asleep, or to quickly remedy night wakings in the middle of the night.

Co-sleeping was common among Black families. In addition to co-sleeping for practical reasons (limited number of beds and bedrooms), parents with adequate room and resources often chose to co-sleep to provide a sense of emotional security for children. Shift in family structure or emotional security were also themes for co-sleeping by choice that emerged from the qualitative data. Bringing child to parents' bed was a consistent theme for how parents remedied night wakings sleep problems. Select quotes below illustrate parents' perspectives on co-sleeping, bedtime routines and sleep timing.

**“***That same child that wants the parent, that wants stability and, we get frustrated with these kids when we are like*, ***OK, you need to*** ***go to sleep cause I'm tired, you know, then where is the bonding****?”-Mother of a 3-year-old*

“***oh, yeah, let's trim this down (laughter). We're down the parent, so let's simplify the*** ***whole process and*** *yeah, love you kisses, let's condense where we can so that it gets back in (laughter).”-Mother of a 5-year-old*

“***maybe if it was like my number one issue or my number one concern*, ***maybe I would be able to do that, but kind of now it's like, OK, it's, it's your grandmother's birthday*. ***Like*,** ***we're not going to get home before eight today*.**”*-Mother of a 4-year-old*

“*...they were going to bed 10:00 or later, whenever they felt like it… S****o um I did notice*** ***that once I started getting them on the schedule of 8:30 it's bedtime, um, she, she started*** ***doing a lot better in 3K, less tantrums and I believe her, she was acting out because she was*** ***tired*, ***so that was one struggle that I dealt with beforehand, but it's a lot better now…* ***because if they're down by 8:30, I can be in bed by 10. Um, and also it's like I'm*** ***not as, I'm not on edge as much um because if I'm not getting sleep, then I'm*** ***also cranky just like they are, so that's, that's definitely a bright spot*.**”*-Mother of a 3-year-old*

“…***we aim for 8:30****, but in all honestly, it probably doesn't happen until, they probably* ***don't get in the bed till like 9:20*.**”*-Mother of a 5-year-old boy*

“*I would prefer otherwise, but um* ***we don't have the necessary um accommodations*,** ***you know, for her to have her own room anything like that, so we have to do it*** ***together*.**”*-Mother of a 6-year-old*

“*I'm happy that they're sleeping through the night when I'm in there. Um, d****oes it suck*** ***that I have to go back and forth week on, week off, from my bed to this air mattress*, ***but at the end of the day, i****f it helps my kids, I'm going to do it*.**”*-Father of a 5-year-old*

#### 3.2.3 Household and home contextual factors

Household structure included both mom and dad being a part of the bedtime routine in two-parent households, with mom who was typically the main decision maker. Grandmothers and aunts were extended family members who were a part of bedtime routines for some children, mostly during weekends. Additional contextual factors in the home related to child's sleep were related to room or bed sharing, light exposure, media use before bed, as well as parent individual knowledge and beliefs about their child's sleep. Select quotes reflect parent responses related to home environment.

“*But yeah* ***When [Child] with [Child] by himself, it's easy to get him to*** ***sleep. But when [Child #2] around, it's it takes longer*** *because I don't know. He's just a boss, I guess (laughter). But um He [Child] does pretty well with sleep, but sometimes he uh, the only thing is sometimes he gets up at night time and he'll come into my bed. I don't know It's hard to get them to stay in their bed throughout the night sometimes and* ***I think like he was doing a little better with before [Child*** ***#2] started coming out the bed too, because now if [Child #2] comes out the bed*** ***and comes out my bed and he wakes up, he he does the same thing****.”-Mother of 6-year-old*

“*[Child]* ***stays asleep, but usually wakes up- [Child] is the older one*, ***I'm sorry. She usually wakes up in the middle of the night to go sleep in my room. And [Child #2] doesn't really do that that stays asleep and otherwise the only reason otherwise they would wake up is to use the restroom. They have been on occasion where* ***they wake up and they play*** *and so you hear it so you're like, OK, go to sleep now but that's not a regular occurrence.”-Mother of a 5-year-old*

“*Um, so, now, so we kind of* ***phased the lamp out*, ***so the lamp was sitting on his desk, then the lamp was sitting on his floor, making its way out. Then it's by the door, then it's outside the door, then it's around the corner*. ***So now his sleep*** ***environment is in the dark, um, door open****. He likes his door open.”-Mother of a 4-year old*

### 3.3 Evidence-based sleep intervention feedback

Parents were presented evidence-based sleep strategies to promote bedtime routines (e.g., ParentCorps routine chart, Sleep Train and Moshi audio-based sleep aide). A few caregivers expressed that they were already carrying out the strategies presented and felt affirmed to see the them included. Incentive-based strategies based on the child receiving a reward for successfully carrying out a sleep strategy were perceived burdensome or ineffective for their child. Some caregivers felt certain strategies may not have been age appropriate or did not align for Black families (e.g., a bedtime pass strategy felt institutionalized like a hall pass). Caregivers who had prior experience with independent sleep strategies found that the approaches may have been effective for them, or felt a more gradual process may be needed for separation. Interactive charts (e.g., sleep train) were the most overwhelmingly received strategy. Parents perceived the chart as very engaging, modifiable and flexible to adjust daily, and provided a sense of accomplishment after completing each task.

## 4 Discussion

Thirty Black parents of young children with mild sleep problems shared their perspective about the contextual definition of sleep health for their family. Barber et al. (2025) conducted a qualitative study to understand perceptions of sleep health among Black American adults. Similarly, their study aimed to identify determinants of poor sleep health and inform tailored interventions among this subpopulation group facing sleep disparities. Focus group participants in their study defined healthy sleep as quality over quantity. Participants recognized the importance of sleep for health and wellbeing (Barber et al., 2025). Our qualitative and quantitative study extends this work to Black families. Our study sample elucidated factors at the individual (e.g., beliefs and knowledge about their child's sleep, household (e.g., family structure), and home environment (e.g., light exposure; bed or room sharing) that influence parents' bedtime routine practices. Our study sample was relatively highly educated compared to other studies that have investigated sleep health among Black children (Guglielmo et al., 2018; Smith et al., 2019), indicating that behavioral sleep challenges persist among relatively socio-economically advantaged Black families. Families practice bedtime routines with their children, but still face behavioral challenges (e.g., bedtime resistance or sleep anxiety) when trying to get their child to sleep. Black families in the present study value connection and bonding, and desire flexibility. Flexibility related to the target bedtime and start of the evening bedtime routine or individual components of the routine (e.g., skipping bath to the morning). Black family sleep health may include co-sleeping, at the sacrifice of the mother's sleep health, who is also typically the main decision-maker at bedtime. Extended family members (e.g., grandma or aunt) may be a part of bedtime practices due to family structure or work schedules.

Qualitative components of our study complemented quantitative data, expanding upon quantitative data responses and explained the context for family sleep behaviors. An example of data divergence can be illustrated by child bedtimes reported in the surveys, and thematic analysis of child bedtimes gleaned from qualitative interviews. Survey responses indicated that 82% of children reported a bedtime before 9 pm, but qualitative results indicated 70% of children went to bed before 9 pm, with a more specific breakdown of about 30% having a bedtime between 7 and 8 pm and 40% have a bedtime between 8 and 9 pm. Similarly, the reasons for delayed bedtime were vividly captured during qualitative interviews (e.g., delay in starting the bedtime routine due to family gatherings or child overstimulation before bedtime due to family play time/activities) that survey data was not able to capture. In contrast, convergent findings across both qualitative and quantitative data collected was the family practice of co-sleeping and having a bedtime routine. All of the families interviewed reported having a bedtime routine as consistently reported in the BRQ, and co-sleeping was a family sleep practice reported in both the CRI and PSIS. Semi-structured interviews allowed parents to explain the reasoning behind co-sleeping practices (e.g., to ameliorate night wakings; parent-child bonding/comfort). The human-centered design empathy interview approach may allow for dialogue that an open-ended response option in a survey may not capture.

Literature suggests that healthy pediatric sleep is marked by consistent bedtime routine, regular sleep timing, with child independent sleep in their own bed (Hale et al., 2011; Kitsaras et al., 2018; Mindell et al., 2017). Our study indicates that healthy sleep in Black families includes a contextual definition that is flexible in sleep timing and bedtime routine activities, and may include co-sleeping. Non-western cultures may welcome communal practices as part of their home, including those related to sleep. For instance, co-sleeping is a common sleep practice in Japanese sleep culture, that has not been linked with sleep problems in early childhood (Latz et al., 1999). To this end, what is appropriate sleep guidance for one group of people, may not be appropriate for another group. Parents may have a sleep time target but actual sleep timing may be later than the targeted time. For instance, a subset of families shared having bedtimes for their young children after 9 pm. The reported reasons for delayed bedtimes included family events, one parent traveling for work, routine inconsistencies, child overstimulation or the child seeking comfort from the parent. As such it is likely not enough to share general recommendations with parents, but future interventions will require a more nuanced approach to behavior change that incorporates parents' values and goals for themselves and their children.

Parents sacrificed their own sleep health for their child's sleep. Barber et al. (2025)'s sample predominantly comprised of Black women, perceived sleep quantity as secondary to quality. Similarly, 90% of our study sample comprised of Black women experiencing insufficient sleep and poor sleep quality. In addition to poor sleep health, 90% of parents reported feeling exhausted after putting their child to sleep. The selflessness of parents to sacrifice their own wellbeing, may be due to Black woman superwoman schema, whereby women put themselves last, at the expense of their own health (Erving et al., 2024). Similarly, the lack of caring for oneself and putting the needs of one's children first, was also evident among Chinese-American parents of young children (Chung et al., 2025). Future behavioral sleep interventions should be tailored to engage both the mother and child dyad. Dyadic interventions should address parent stress and promote child task-shifting and sleep independence.

Black families were creative in developing their own interactive routine charts and visual cues for their children (e.g., large check-list charts; visual traffic light toys) in effort to inculcate child independence with bedtime routine tasks and transitions. Incentives were not received as a favorable parenting practice to inculcate positive bedtime routine behavior adoption for their child. Incentives were perceived as burdensome or an ineffective parenting strategy. Similarly, some evidence-based routine charts that included a “Who” column to designate the individual responsible for each bedtime task were interpreted as tools that could help children's ownership of responsibilities or involve another parent, such as fathers, in the bedtime routine. Child independence or engaging another parent may be one way to alleviate burden on the primary parent.

### 4.1 Limitations

Study recruitment mostly held in the New York tri-state area limits the external generalizability to the broader population of Black families. First author, AC, completed 29 of the 30 interviews and captured the geographic location of parents via virtual interviews if it was mentioned during the interview (e.g., a family that recently moved to Washington, DC, describing their child's adjustment to a sleep environment in a new home). Unfortunately, we did not formally capture data asking participants where they were located geographically. Additionally, while our sample size meets the criteria of for a phenomenology approach, future research may consider a larger sample size with statistical power for statistical analysis or UX design. Additionally, our study only had one touch point, limiting the ability to study our sample over a prolonged period of time to identify meaningful relationships and patterns, per phenomonological inquiry. Selection bias may have also been introduced among the study sample given the relatively small sample size.

## 5 Conclusion

Our study indicates that both qualitative and quantitative data may be needed to gather a full picture of parent experiences during their child's bedtime. Quantitative data alone may not be sufficient. Qualitative data allows for understanding of contextual factors that may affect the reality of a child's bedtime routine. Survey responses may be subject to social desirability and response bias, where parents may be concerned about judgement or may not accurately recall actual bedtimes without talking through the bedtime routine steps, in the way that an interview lends it'self to during a conversation.

Among our sample of Black families, mostly from the New York area, sleep health among Black families of young children includes bedtime routines that seek child independence, preserve bonding time, and reduce the stressful process of getting their child to bed. Co-sleeping was a common sleep practice. Parents sacrifice their own sleep and wellbeing for their child's sleep health. Insights from this study may inform the development of a new framework that considers the home and individual factors that affect sleep among Black families. Future research may include the development of a tailored intervention to promote sleep health in Black families with young children.

## Data Availability

The raw data supporting the conclusions of this article will be made available by the authors, without undue reservation.
